# Hydrogen Sulfide Prevents Hydrogen Peroxide-Induced Activation of Epithelial Sodium Channel through a PTEN/PI(3,4,5)P_3_ Dependent Pathway

**DOI:** 10.1371/journal.pone.0064304

**Published:** 2013-05-31

**Authors:** Jianing Zhang, Shuo Chen, Huibin Liu, Bingkun Zhang, Ying Zhao, Ke Ma, Dan Zhao, Qiushi Wang, Heping Ma, Zhiren Zhang

**Affiliations:** 1 Departments of Clinical Pharmacy and Cardiology, the 2nd Affiliated Hospital, Harbin Medical University, Key Laboratories of Education Ministry for Myocardial Ischemia Mechanism and Treatment, Harbin, P.R. China; 2 Department of Physiology, Emory University School of Medicine, Atlanta, Georgia, United States of America; University of Pittsburgh, School of Medicine, United States of America

## Abstract

Sodium reabsorption through the epithelial sodium channel (ENaC) at the distal segment of the kidney plays an important role in salt-sensitive hypertension. We reported previously that hydrogen peroxide (H_2_O_2_) stimulates ENaC in A6 distal nephron cells via elevation of phosphatidylinositol 3,4,5-trisphosphate (PI(3,4,5)P_3_) in the apical membrane. Here we report that H_2_S can antagonize H_2_O_2_-induced activation of ENaC in A6 cells. Our cell-attached patch-clamp data show that ENaC open probability (*P_O_*) was significantly increased by exogenous H_2_O_2_, which is consistent with our previous finding. The aberrant activation of ENaC induced by exogenous H_2_O_2_ was completely abolished by H_2_S (0.1 mM NaHS). Pre-treatment of A6 cells with H_2_S slightly decreased ENaC *P_O_*; however, in these cells H_2_O_2_ failed to elevate ENaC *P_O_*. Confocal microscopy data show that application of exogenous H_2_O_2_ to A6 cells significantly increased intracellular reactive oxygen species (ROS) level and induced accumulation of PI(3,4,5)P_3_ in the apical compartment of the cell membrane. These effects of exogenous H_2_O_2_ on intracellular ROS levels and on apical PI(3,4,5)P_3_ levels were almost completely abolished by treatment of A6 cells with H_2_S. In addition, H_2_S significantly inhibited H_2_O_2_-induced oxidative inactivation of the tumor suppressor phosphatase and tensin homolog (PTEN) which is a negative regulator of PI(3,4,5)P_3._ Moreover, BPV_(pic)_, a specific inhibitor of PTEN, elevated PI(3,4,5)P_3_ and ENaC activity in a manner similar to that of H_2_O_2_ in A6 cells. Our data show, for the first time, that H_2_S prevents H_2_O_2_-induced activation of ENaC through a PTEN-PI(3,4,5)P_3_ dependent pathway.

## Introduction

The epithelial sodium channel (ENaC) mediates Na^+^ absorption across epithelial cells in the kidney collecting duct, lung, distal colon, and sweat duct. Na^+^ transport is critical for the maintenance of Na^+^ homeostasis and thus plays a critical role in maintenance of salt balance and systemic blood pressure. Overactivation of ENaC causes hypertension, as seen in Liddle’s syndrome [1]. However, it remains unclear whether enhanced ENaC activity accounts for salt-sensitive hypertension. In salt-sensitive animal model, high salt intake leads to an increase in the production of reactive oxygen species (ROS) including superoxide (O_2_^−^) [2] and H_2_O_2_ [3] in the kidney by stimulating NAD(P)H oxidase [4]. ROS play an important role in signal transduction and have been shown to influence ENaC activity. One example is that aldosterone can regulate ENaC activity by elevating superoxide (O_2_^−^) production in A6 cells [5].

Our recent study shows that H_2_O_2_ stimulates ENaC in A6 distal nephron cells via elevation of PI(3,4,5)P_3_ [6], which is known to stimulate ENaC [7]. PI(3,4,5)P_3_, the product of the phosphorylation of phosphatidylinositol 4,5-bisphosphate (PI(3,4,5)P_2_) by phosphoinositide 3-kinase (PI3K), plays an important role in transducing signals from growth factors, hormones and other extracellular activators to intracellular pathways. The tumor suppressor phosphatase and tensin homolog (PTEN), a lipid phosphatase, reduces the cellular concentration of PI(3,4,5)P_3_ and acts as a negative regulator of PI3K signaling pathways [8]. Thus, loss of PTEN activity would result in the accumulation of PI(3,4,5)P_3_. It has been proposed that inactivation of PTEN by H_2_O_2_ might be necessary to increase the abundance of PI(3,4,5)P_3_ and subsequent activation of Akt [9], [10].

Hydrogen sulfide (H_2_S), as an important intracellular and intercellular gaseous messenger molecule, regulates multiple physiological and pathological processes, including vascular relaxation [11], angiogenesis [12], ischemia/reperfusion (I/R) injury of the heart [13],glucose homeostasis [14] and the function of ion channels [15]. Accumulating evidence demonstrates that H_2_S exerts protective effects against a number of injuries in many organs. One of the main mechanisms responsible for H_2_S protection is antioxidation, not only by enhancing reduced glutathione (GSH, a major cellular antioxidant) [16], but also by directly scavenging superoxide anions [17], H_2_O_2_ [18] and peroxynitrite [19] to suppress oxidative stress. We therefore hypothesize that H_2_S may exert a protective effect against H_2_O_2_-induced ENaC activity in A6 cells. However, to our knowledge, the direct regulation of ENaC activity by H_2_S has never been demonstrated. The A6 cell line, derived from the distal nephron of *Xenopus* kidney, is an established system for *in vitro* study of ENaC regulation. In the present study, we show that H_2_S reverses H_2_O_2_-induced aberrant activation of ENaC by diminishing oxidized PTEN.

## Materials and Methods

### Cell Culture

A6 cells are an established renal cell line derived from the distal segment of *Xenopus* laevis nephron, which is an appropriate cell model for studying ENaC. A6 cells, purchased from American Type Culture Collection (Rockville, MD, USA), were grown in the medium consisting of 2 parts of DMEM/F-12 (1∶1) medium (Invitrogen, USA), 1 part of H_2_O, 15 mM NaHCO_3_ (total Na^+^ = 101 mM), 2 mM L-glutamine, 10% fetal bovine serum (Invitrogen, USA), 25 units/ml penicillin, 25 units/ml streptomycin, as previously described [6]. A6 cells were cultured in plastic flasks in the presence of 1 µM aldosterone at 26°C and 4% CO_2_. After the cells became 70% confluent in the plastic flasks, they were subcultured on the polyester membrane of either *Transwell* inserts (Corning Costar Co, USA) for confocal microscopy assays or *Snapwell* inserts (Corning Costar Co, USA) for cell-attached patch-clamp analysis. To facilitate polarization cells were cultured for at least two to three weeks before accessing experiments.

### Patch-clamp Recording

ENaC single-channel currents were recorded using cell-attached patch-clamp configuration with an Axopatch-200B amplifier (Axon Instruments, USA) as described in our previous works [20], [21]. A6 cells were thoroughly washed with NaCl solution containing 100 mM NaCl, 3.4 mM KCl, 1 mM CaCl_2_, 1 mM MgCl_2_, and 10 mM HEPES, adjusted pH to 7.4 with NaOH. Borosilicate glass electrodes had tip resistances of 7–10 MΩ when filled with NaCl solution. Experiments were conducted at room temperature (22–25°C). The data were acquired by application of 0 mV pipette potential and were sampled at 5 k Hz and low-pass filtered at 1 k Hz with Clampex 10.2 software (Molecular Devices, Sunnyvale, CA, USA). Prior to analysis, the single-channel traces were further filtered at 30 Hz. The total number of functional channels in the patch was determined by observing the number of peaks detected on the current amplitude histograms during at least 10-min recording period. The open probability (*Po*) of ENaC before and after application of chemicals was calculated using Clampfit 10.2 (Molecular Devices, Sunnyvale, CA, USA). Control ENaC activity was recorded at 2 min after forming the cell-attached mode when the ENaC activity had stabilized. We usually recorded at least 30 min before any experimental manipulation in the same patch.

### Stable Transfection of a6 Cells with Enhanced Green Fluorescence Protein-tagged Pleckstrin Homology Domain of Akt (EGFP-PH-Akt)

To detect intracellular PI(3,4,5)P_3_ levels, A6 cells were cultured on the polyester membrane of *Transwell* inserts at a high density to allow the cells to be confluent within three days [6]. Confluent A6 cells were treated for 20 min with Ca^2+^-free and Mg^2+^-free PBS (DPBS, Invitrogen, USA), which was modified with H_2_O (3 parts of PBS with 1 part of H_2_O) to match the osmolarity of amphibian cells. A6 cells were incubated with transfection reagent containing EGFP-PH-Akt DNA construct and Lipofectamin 2000 (Invitrogen, USA) for six hrs and then incubated with regular culture medium for one day. The EGFP-PH-Akt DNA construct contains a geneticin (G418) resistance gene and the transfected cells were continuously cultured, in the presence of 600 µg/mL G418. Four weeks after transfection, cells were ready for assessing further experiments.

### Confocal Laser Scanning Microscopy

Studies were performed using confocal microscopy (Olympus Fluoview1000, Japan) as previously described [6]. Prior to experiments, A6 cells were washed twice with NaCl solution. Immediately following each experimental manipulation, the polyester membrane that supports the A6 cell monolayer was quickly excised and mounted on a glass slide with a drop of NaCl solution to keep the cells alive. Confocal microscopy XY or XZ scanning of A6 cells was accomplished within five min. XY optical sections were performed to provide a flat view of the cells near the apical membrane, across the lateral membrane, or near the basal membrane. XZ optical sections were also performed to provide a lateral view of the cells. In each set of experiments, images were taken using the same parameter settings. The fluorescent intensity of GFP-PH-Akt represents the levels of PI(3,4,5)P_3_ near the apical compartment of the cell membrane. The fluorescent intensity was measured in a randomly selected field including a group of cells by setting the amplitude of the Z-step as 9.5±0.5 µM from the basolateral membrane. Average fluorescent intensity of an individual experiment was obtained as follow: fluorescent intensity measured from a group of cells divided by the number of cells in the randomly selected field.

### Detection of Intracellular Reactive Oxygen Species (ROS) by Confocal Microscopy

A6 cells grown on polyester membrane of *Transwell* inserts were loaded with 2.5 µM 5-(and-6)-carboxy-2′,7′-dichlorodihydrofluorescein diacetate (carboxy-H_2_DCFDA), a membrane-permeable ROS-sensitive fluorescent probe (Invitrogen, USA), which became fluorescent when oxidized. Prior to application of exogenous H_2_O_2_, A6 cells were treated by an iron chelator, 50 µM 2,2′-dipyridyl for three min [22]. Labeled cells were washed twice in modified DPBS before analyzed by confocal microscopy. ROS levels were represented with fluorescence intensity.

### Western Blot Analysis

The expression of PTEN protein was examined using western blot experiments. Cells were incubated at 26°C in the medium with or without NaHS. Cells were then harvested and total protein was extracted. Cell lysate was loaded and electrophoresed on a 10% SDS-polyacrylamide gel with running buffer and transferred to polyvinylidene fluoride (PVDF) membranes. After one hr of blocking with 5% nonfat dry milk in phosphate-buffered saline (PBS), membranes were incubated with primary antibody specific to PTEN (Abcam, UK; 1∶500 dilution) or horseradish peroxidase-conjugated β-actin (Santa Cruz Biotechnology, USA; 1∶5000 dilution) overnight with gentle agitation at 4°C. The next day, the membrane was incubated with a horseradish peroxidase-conjugated secondary antibody (Santa Cruz Biotechnology, USA; 1∶5000 dilution) for one hr at room temperature. The membranes were developed using an enhanced chemiluminescence (ECL) kit (Invitrogen, USA) and scanned densitometry (Bio-Rad, USA). The band densities were quantified by densitometry using Quantity One Software and the image densities were normalized to densities measured in control samples.

### Detection of Oxidized PTEN

Cells subjected to a various treatments including an iron chelator, 50 µM 2,2′-dipyridyl were scraped into 0.2 ml of ice-cold 50% trichloroacetic acid and transferred to microfuge tubes. The cell suspensions were sonicated briefly and then centrifuged at 2000 g for 5 min at 4°C. The supernatants were removed, and the pellets were washed with cold acetone twice and then solubilized in 0.2 ml of 100 mM Tris-HCl (pH 6.8) buffer containing 2% SDS and 40 mM n-ethylmaleimide (NEM; Sigma, USA). Portions (10 µl) of the solubilized pellets were subjected to SDS-PAGE under non-reducing conditions (without DTT) and transferred to a PVDF membrane. The membrane was then treated with PTEN specific antibody as ascribed above. Reduced and oxidized forms of PTEN were detected as described previously [10].

### Chemicals and Reagents

Unless otherwise noted, all chemicals and reagents were purchased from Sigma Aldrich (St. Louis, MO. USA). H_2_O_2_ was purchased from Fisher Scientific (USA) and was diluted with NaCl solution prior to assessing experiments.

### Data Analysis

Data are presented as mean ± S.E. Statistical analysis was performed with SigmaPlot and SigmaStat Software (Jandel Scientific, CA. USA). Paired *t* test or student *t* test was used for comparisons between pre- and post-treatment activities. Analysis of variance was used for multiple comparisons among various treatment groups. Differences were considered statistically when *P*<0.05.

## Results

### H_2_O_2_-induced Enhancement of ENaC Activity is Reversed by H_2_S in A6 Cells

**Figure 1:**
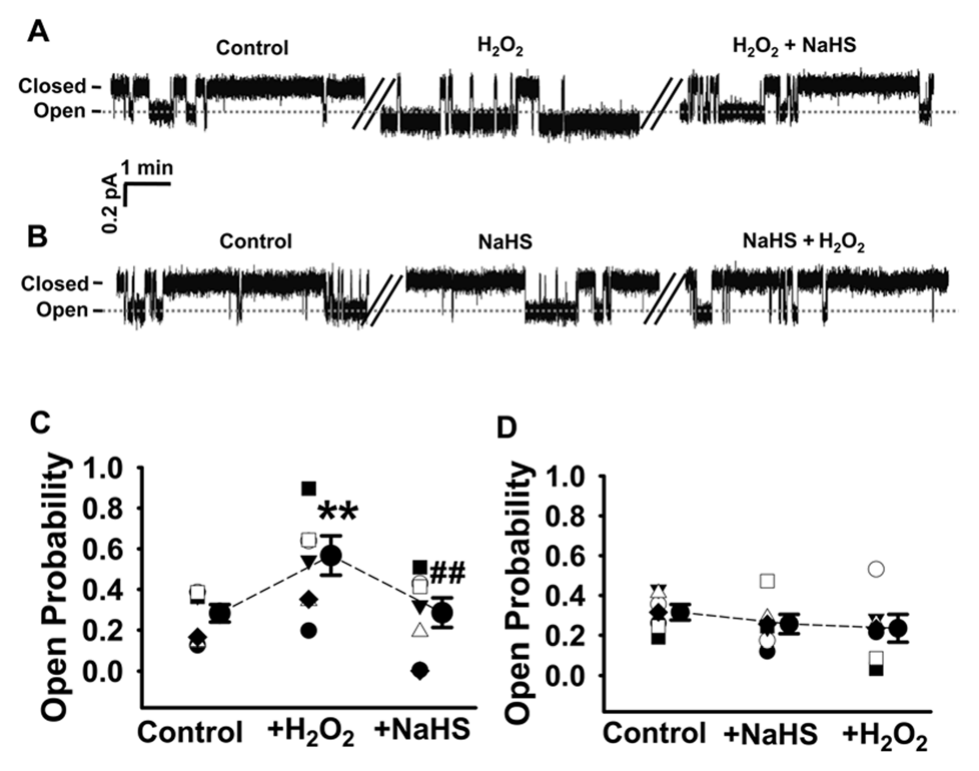
H_2_S inhibits H_2_O_2_-induced enhancement of ENaC activity in A6 cells. (A) and (B) Representative single-channel ENaC traces respectively recorded from two A6 cells before and after addition of 3 mM H_2_O_2_ and 3 mM H_2_O_2_+0.1 mM NaHS to the basolateral bath; the breaks between the traces indicate omitted 20 min of the recording periods; downward events represent channel openings (the dashed gray line); the currents were monitored for at least 30 min before and after chemical application for both in (A) and (B). (C) and (D) Summarized *Po* of ENaC before and after application of different reagents as indicated (n = 6 paired experiments). **P<0.01 compared with control group, ^##^ *P*<0.01 compared with H_2_O_2_ treatment group.

To investigate the effect of H_2_S on H_2_O_2_-induced enhancement of ENaC activity, cell-attached patch-clamp technique was employed. Single-channel current of ENaC was recorded for about 90 min in each experiment. Since the responses had a long latency, in the figures we omitted a period of record and only showed the representative recordings for about six min before and after application of either H_2_O_2_ or NaHS (a donor of H_2_S) to the cells. Consistent with our previous findings, addition of 3 mM H_2_O_2_ to the basolateral bath led to a significant increase both in ENaC *Po* from 0.28±0.04 to 0.57±0.10 (*P*<0.01; n = 6) and in the single-channel amplitude of ENaC (Figure 1A) [6]. In the presence of H_2_O_2_, application of 0.1 mM NaHS to the basolateral bath led to a significant decrease in ENaC *Po* from 0.57±0.10 to 0.29±0.07 (*P*<0.01; n = 6) (Figure 1A and C). In contrast, application of 0.1 mM NaHS to the basolateral bath slightly decreased ENaC *Po* from 0.32±0.04 to 0.26±0.05 (*P*>0.05; n = 6); interestingly, H_2_O_2_ failed to increase ENaC *Po* in the cells pretreated with 0.1 mM NaHS (0.32±0.04 under control condition vs. 0.26±0.05 with NaHS vs. 0.24±0.07 with NaHS+H_2_O_2_; *P*>0.05; n = 6) (Figure 1B and D). These results suggest that H_2_S exerts a strong protective effect on H_2_O_2_-induced enhancement of ENaC activity in A6 cells.

### H_2_S Attenuates Exogenous H_2_O_2_-induced Oxidative Stress in A6 Cells

**Figure 2:**
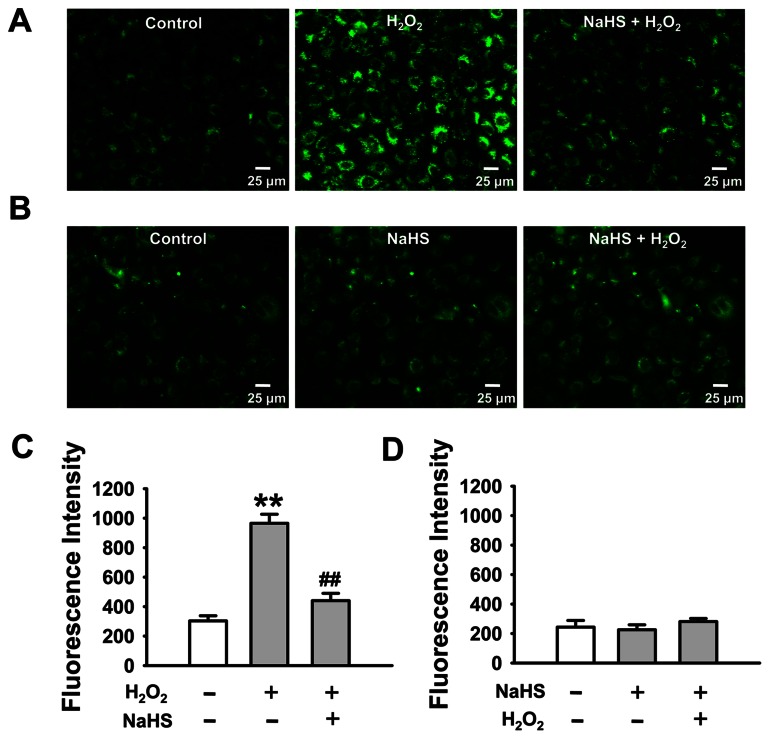
H_2_S ameliorates H_2_O_2_-elicited oxidative stress in A6 cells. All experiments were assessed in the presence of 50 µM 2,2′-dipyridyl. The images represent the level of intracellular ROS detected by the membrane-permeable fluorescent probe, carboxy-H_2_DCFDA, under indicated conditions in A6 cells. (A) The left image shows that there was a certain residual level of intracellular ROS under control condition; middle image shows a significant elevation of intracellular ROS level upon application of 3 mM H_2_O_2_; right image shows that this H_2_O_2_-induced increase in intracellular ROS was abolished by additional 0.1 mM NaHS. (B) The images represent that the intracellular ROS levels under control (left), treatment of A6 cells with 0.1 mM NaHS for 30 min (middle) followed by additional 0.1 mM H_2_O_2_ for 30 min (right). (C) and (D) Summarized fluorescent intensity of (A) and (B), respectively. Data were from three independent paired experiments. **P<0.01 compared with control group, ^##^ *P*<0.01 compared with H_2_O_2_ treatment group.

Inhibition of oxidative stress is thought to account for the cardioprotective effects of H_2_S during ischemia/reperfusion (I/R) [13], [23]. However, it is unknown whether H_2_S could reduce H_2_O_2_-induced oxidative stress in A6 cells. Therefore, the levels of intracellular reactive oxygen species (ROS) were examined with a fluorescent probe, DCF (refer to Materials and Methods) in the presence of H_2_O_2,_ H_2_O_2_+ NaHS, or NaHS, respectively. We performed experiments in the presence of an iron chelator, 50 µM 2,2′-dipyridyl, to confirm whether oxidation is due to Fenton chemistry, where we treated the cells with 2,2′-dipyridyl for three min followed by exposing to H_2_O_2_ for 30 min. Our results show that exposure of A6 cells to H_2_O_2_, in the presence of 2,2′-dipyridyl, induced significant accumulation of intracellular ROS levels (Figure 2A and 2C; the same as seen in the absence of 2,2′-dipyridyl). These results suggest that exogenous H_2_O_2_-induced oxidation is not due to Fenton chemistry. Furthermore, in the cells pretreated with 0.1 mM NaHS for 30 min, addition of 3 mM H_2_O_2_ failed to increase intercellular ROS (Figure 2B and D). We have carried out the MTT experiments to detect whether H_2_S affects cell viability (Text S1). The MTT assay showed that H_2_S at 0.05, 0.1 and 0.3 mM had no effect on cell viability (Figure S1). These results suggest that H_2_S can reverse H_2_O_2_-induced accumulation of intracellular ROS.

### H_2_S Diminishes H_2_O_2_-induced Elevation of PI(3,4,5)P_3_ Near the Apical Compartment of A6 Cells

**Figure 3:**
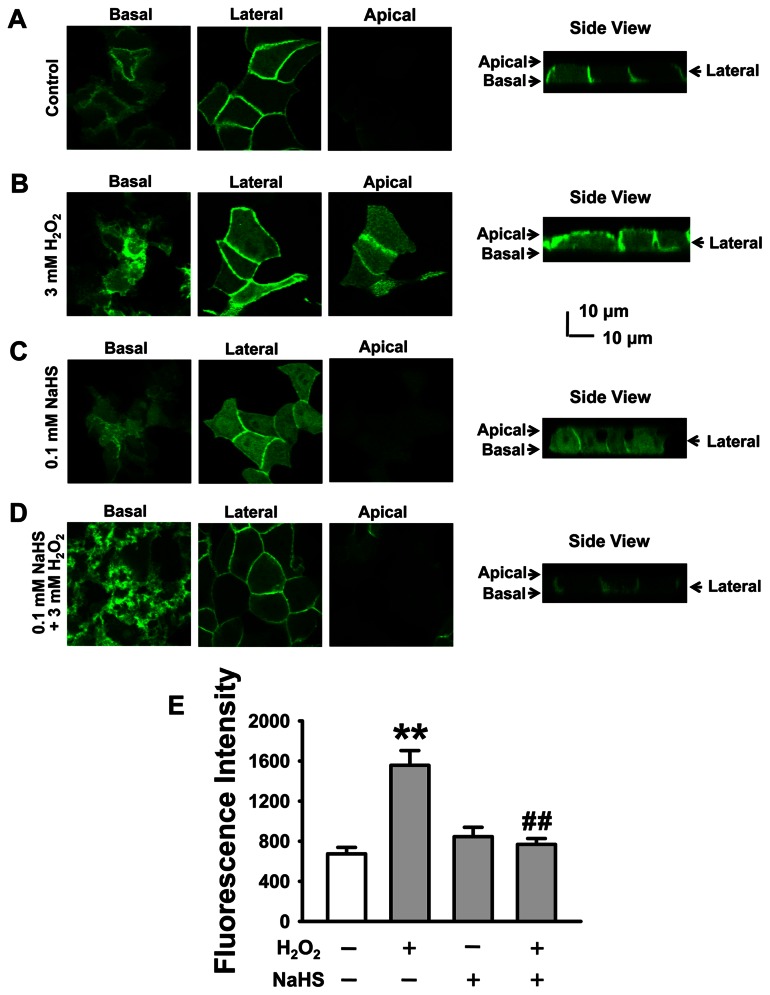
H_2_S inhibits H_2_O_2_-induced elevation of PI(3,4,5)P_3_ near the apical compartment of A6 cells. Left images show confocal microscopy XY sections of apical, lateral, and basal membranes of A6 cells as indicated. Right images show XZ sections of A6 cells. (A) Control; (B) the cells were treated with 3 mM H_2_O_2_ for 30 min; (C) the cells were treated with 0.1 mM NaHS for 30 min; (D) the cells were co-treated with 3 mM H_2_O_2_+0.1 mM NaHS for 30 min. (E) Summarized mean fluorescent intensities measured in and in the vicinity of the apical membrane from three independent experiments, which represent the levels of PI(3,4,5)P_3_ near the apical region of the membrane. **P<0.01 compared with control group, ^##^ *P*<0.01 compared with H_2_O_2_ treatment group.

We have recently shown that H_2_O_2_ stimulates ENaC via elevation of PI(3,4,5)P_3_ near the apical compartment of the cell membrane [6]. Therefore, we reasoned that NaHS may diminish exogenous H_2_O_2_-induced enhancement of ENaC activity via reducing accumulation of PI(3,4,5)P_3_ near the apical compartment of A6 cells. To test this hypothesis, the cells were stably transfected with the EGFP-PH-Akt construct containing the PH domain of Akt which selectively binds to PI(3,4,5)P_3_. Consistent with our previous studies [24], under control conditions PI(3,4,5)P_3_ was detected mainly in the lateral and basal membranes (Figure 3A and D); treatment of the cells with 3 mM H_2_O_2_ for 30 min resulted in a dramatic elevation of PI(3,4,5)P_3_ near the apical compartment of cell membrane (Figure 3B and E). In contrast, when the cells were treated with 0.1 mM NaHS or co-treated with 0.1 mM NaHS and 3 mM H_2_O_2_ for 30 min, there was no significant accumulation of PI(3,4,5)P_3_ near the apical compartment of cell membrane (Figure 3C–E). The fluorescent intensity, measured in and in the vicinity of the apical membrane, was used as a readout of PI(3,4,5)P_3_ levels. The data shown in Figure 3E represent the mean value of PI(3,4,5)P_3_ in or near the apical membrane of three independent experiments. These data suggest that H_2_S prevents H_2_O_2_-induced elevation of PI(3,4,5)P_3­_ in or near the apical membrane. We have also carried out the confocal experiments to detect the PI(3,4,5)P_3_ levels near the apical compartment of membrane in the presence of 0.3 mM, H_2_O_2_ (Figure S2). We found that there is a slight, but significant increase in PI(3,4,5)P_3_ level near the apical compartment of membrane, albeit required a longer treatment time (one hr).

### H_2_S Ameliorates H_2_O_2_-induced Inactivation of PTEN

**Figure 4:**
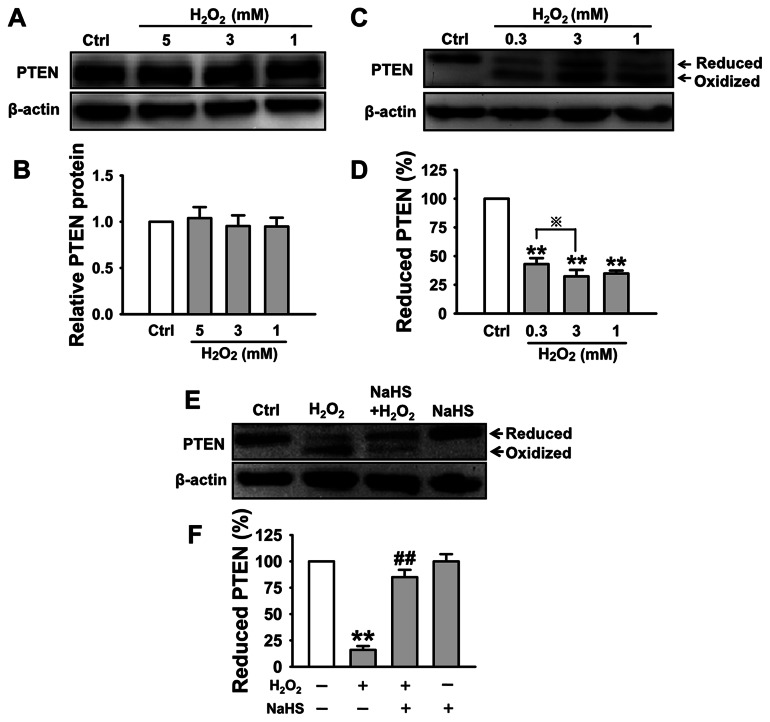
H_2_O_2_ oxidizes PTEN and H_2_S protects PTEN to be oxidized by H_2_O_2_. All experiments were assessed in the presence of 50 µM 2,2′-dipyridyl. (A) and (B) Western blot analysis demonstrates that the total PTEN protein expression was not altered by treatment of A6 cells with 1 mM, 3 mM and 5 mM H_2_O_2_. (C) and (D) Western blot analysis shows that the magnitude of reduced (active) PTEN was dramatically decreased upon treatment of A6 cells with 0.3 mM, 1 mM and 3 mM H_2_O_2_ for 30 min; the intensity of the bands corresponding to reduced PTEN in the top panels of (C) was determined and presented as a percentage of the sum of the intensities of the bands corresponding to the oxidized and reduced proteins. (E) and (F) show that H_2_S protected 3 mM H_2_O_2_ induced PTEN oxidation (n = 3 in each group) **P<0.01 compared with control group; 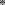 *P*<0.05 compared with 0.3 mM H_2_O_2_ treatment group;^ ##^ *P*<0.01 compared with H_2_O_2_ treatment group.

PI(3,4,5)P_3_ levels are reciprocally controlled by PI3K and the lipid phosphatase PTEN. PTEN negatively regulates the PI3K signaling via dephosphorylation of PI(3,4,5)P_3_ to PI(3,4,5)P_2_. It has been reported that ROS can inactivate PTEN [25] and that H_2_O_2_ oxidizes PTEN within its catalytic domain by forming a disulfide bond between Cys124 and Cys71 in the active site, thus inactivating its phosphatase function [9]. Therefore, we tested whether inactivation of PTEN account for H_2_O_2_-induced elevation of PI(3,4,5)P_3_. Although western blot data demonstrated that total PTEN expression in the cells treated with H_2_O_2_ for 30 min was not altered (Figure 4A and B), however, the abundance of reduced PTEN (active PTEN) was dramatically decreased in the cells respectively treated with 0.3 mM, 1 mM and 3 mM H_2_O_2_ (Figure 4C and 4D). Oxidized PTEN (inactive PTEN) has two less cysteine residues available for alkylation, which results in a lower molecular weight form of the protein. Our results indicated that the abundance of oxidized (inactivated) PTEN in H_2_O_2_-treated cells was significantly greater, compared to that in the cells co-treated with both NaHS and H_2_O_2_ (Figure 4E and F). Oxidized PTEN was not detected in either control cells or in the cells pretreated with NaHS alone (Figure 4C, E and F). These results suggest that H_2_S almost completely reverses H_2_O_2_-induced inactivation of PTEN, which accounts, at least in part, for the inhibitory effect of H_2_S on accumulation of PI(3,4,5)P_3_ in the apical membrane of A6 cells.

### Inhibition of PTEN Leads to Accumulation of PI(3,4,5)P_3_ Near the Apical Compartment of Membrane

**Figure 5:**
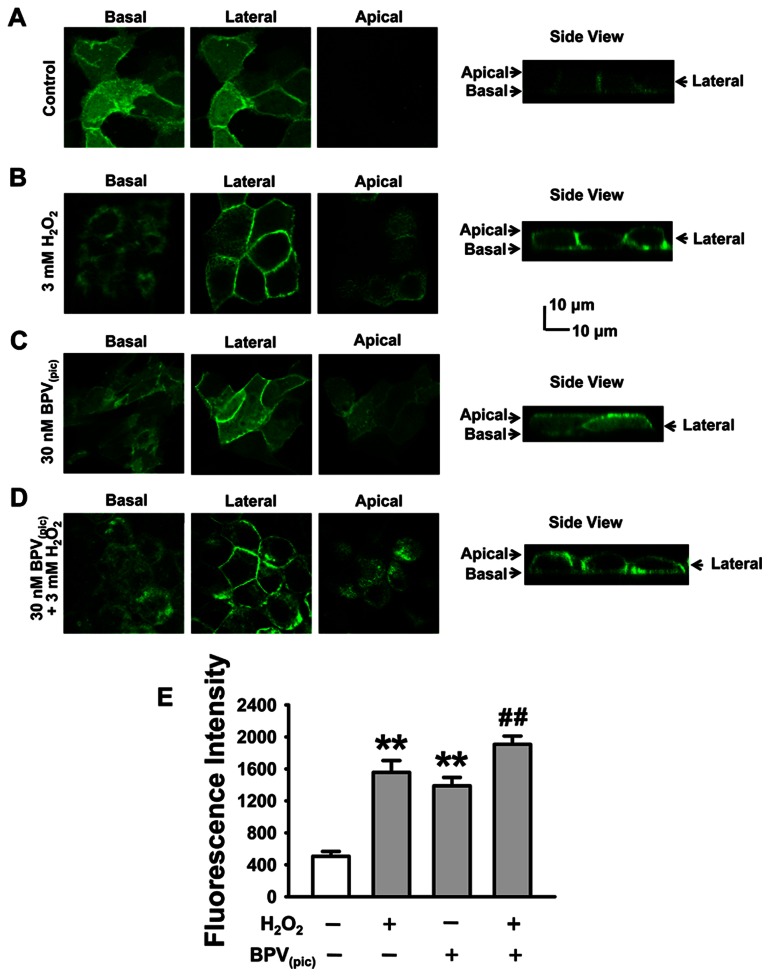
Inhibition of PTEN by BPV_(pic)_ results in accumulation of PI(3,4,5)P_3_ near the apical compartment of A6 cells. The experiments were performed under different conditions as follows: (A) control; (B) the cells were treated with 3 mM H_2_O_2_ for 30 min; (C) the cells were treated with 30 nM BPV_(pic)_ for 30 min; (D) the cells were co-treated with 30 nM BPV_(pic)_ +3 mM for 30 min. (E) Summarized mean fluorescent intensities measured as ascribed in Figure 3 from three independent experiments, which represent the level of PI(3,4,5)P_3_ in the apical membrane. **P<0.01 compared with control group, ^##^ *P*<0.01 compared with BPV_(pic)_ treatment group.

To further confirm activation of PTEN accounts for the cellular distribution of PI(3,4,5)P_3_, 30 nM BPV_(pic)­_ (dipotassium bisperoxo(pyridine-2-carboxyl)oxovanadate; ENZO, USA), a specific PTEN inhibitor, was used to treat A6 cells since it has an IC_50_ of 20–40 nM [26]. Under control conditions PI(3,4,5)P_3_ was mainly located at the basal and lateral membrane (Figure 3A and 5A). In contrast, the PI(3,4,5)P_3_ levels near the apical compartment of the cell membrane were significantly increased in A6 cells exposed to BPV_(pic)_, to the same degree as seen in 3 mM H_2_O_2_ treated cells (Figure 5B, C and E). Compared to the cells treated with BPV_(pic)_ alone, treatment of the cells with both BPV_(pic)_ and H_2_O_2_ induced a slightly, but significantly additive effect on PI(3,4,5)P_3_ levels, probably due to additional activation of PI3K by H_2_O_2_ (Figure 5B–E). These results confirm that PTEN activity accounts for accumulation of PI(3,4,5)P_3_ near the apical compartment in A6 cells.

### Inhibition of PTEN Enhances ENaC *P_O_*

**Figure 6:**
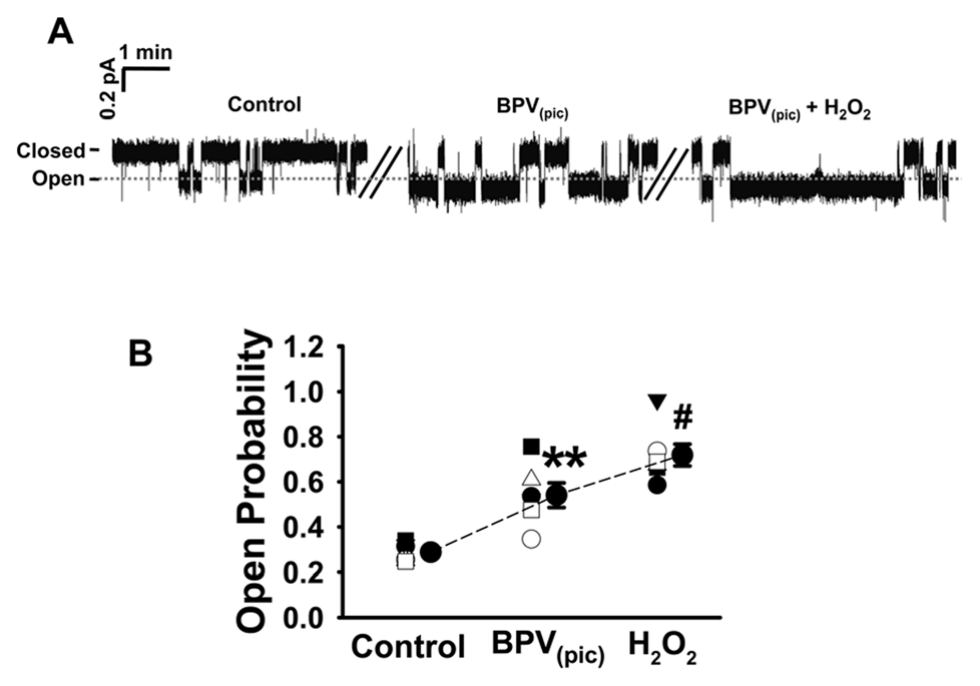
Inhibition of PTEN leads to an aberrant activation of ENaC in A6 cells. Representative single-channel ENaC trace recorded from an A6 cell before and after addition of 30 nM BPV_(pic)_ and 3 mM H_2_O_2_ to the basolateral bath. Two breaks between the traces indicate the 20 min omitted recording periods. (B) Summarized *Po* of ENaC before and post application of different reagents. n = 5 paired experiments. **P<0.01 compared with control group, ^#^ *P*<0.05 compared with BPV_(pic)_ treatment group.

Since BPV_(pic)_ caused increases in PI(3,4,5)P_3_ levels, in the following experiments we tested whether application of BPV_(pic)_ to A6 can alter ENaC *P_O_*. As shown in Figure 6, ENaC *Po* was significantly increased from 0.29±0.02 to 0.56±0.04 by application of BPV_(pic)_ to the apical bath (*P*<0.01; n = 6). In parallel with the effects on PI(3,4,5)P_3_ levels, treatment of the cells with both BPV_(pic)_ and 3 mM H_2_O_2_ further increased ENaC *Po* from 0.56±0.04 to 0.70±0.04 (Figure 6; *P*<0.05; n = 6). Since H_2_O_2_ elevates PI(3,4,5)P_3_ both by activating PI3K and by inactivating PTEN, we argue that this synergistic effect of BPV_(pic)_ and H_2_O_2_ on ENaC activity is probably due to H_2_O_2_ -induced activation of PI3K [6].

## Discussion

It is well documented that oxidative stress is one of the main causes of salt-induced kidney injury which might be an important mechanism of salt-sensitive hypertension [3], [4]. Recently, we have shown that H_2_O_2_ stimulates ENaC [6], which plays a key role in maintaining Na^+^ homeostasis and consequently controls systemic blood pressure. H_2_S, an endogenous gaseous mediator, exerts various physiological and physiopathological effects *in vivo*, including anti-oxidative stress and anti-inflammatory response in heart, liver, kidney and other organs [27], [28], [29]. In this study, we show that H_2_O_2_ increases ENaC activity by elevating PI(3,4,5)P_3_ near the apical compartment of A6 cells via both activation of PI3K and inactivation of PTEN. Moreover, our data suggest that as an antioxidant, H_2_S attenuates H_2_O_2_-induced aberrant activation of ENaC in distal nephron cells by reducing PTEN oxidation.

Our previous study showed that high exogenous concentration of H_2_O_2_ is required to counteract the high expression level of catalase in A6 cells, in order to elevate intracellular H_2_O_2_, and to regulate ENaC [6]. We found that exogenous H_2_O_2_ does not significantly elevate intracellular ROS until the concentration of exogenous H_2_O_2_ was in the millimolar range. However, H_2_O_2_, even at 10 mM, did not result in either cell lysis or apoptosis in A6 cells [6], suggesting that the concentrations of H_2_O_2_ used for the current study (3 mM) should not result in any nonspecific effects due to cellular damage. Therefore, such high concentrations of H_2_O_2_ can be used as a tool to manipulate the levels of intracellular H_2_O_2_ in A6 cells and to investigate the mechanisms by which ROS stimulates ENaC. Furthermore, 0.1–0.3 mM H_2_O_2_ induced a similar result in mpkCCD_14_ cells (a mouse cortical collecting duct cell line) as seen in A6 cells (unpublished observations), with much shorter latency. Moreover,treatment of A6 cells with 0.3 mM H_2_O_2_ for six hrs significantly increased ENaC *P_O_* (data not shown). These results are consistent with the notion that there is a high catalase activity in A6 cells.

The H_2_S level can be up to 0.1 mM in human blood [30] and is about 1.6 nmol/mg in intact kidney of rats [31]. Different concentrations of H_2_S (NaHS) (0.2 mM, 0.4 mM, 0.8 mM or even higher concentration) have been used in the previous *in vitro* studies [32], [33]. A recent study showed that NaHS treatment can protect human umbilical vein endothelial cells and fibroblasts against ischemia-reperfusion (I/R)-induced apoptosis [30]. In the present study, we selected the concentrations of 0.1 mM which is more proximate to its physiological levels in human blood. More importantly, our data show that hydrogen sulfide at concentration up to 0.3 mM had no effect on cell viability.

We should note that the inhibitory effect of H_2_S on ENaC activity exhibited a long latency (∼20 min). This slow effect of NaHS is consistent with what has been reported previously [34], where the protective effect of H_2_S on ischemia-reperfusion induced injury, in human umbilical vein endothelial cells, required at least 20 min pretreatment of these cells with 0.1 mM NaHS. Previous studies show that H_2_S readily scavenges CoCl_2_-induced overproduction of ROS in PC12 cells [32], [33]. We found that exogenous H_2_O_2_-induced accumulation of intracellular ROS in A6 cells was significantly abrogated by pretreatment of the cells with NaHS. We can not rule out the possibility that NaHS (H_2_S) may carry out its cytoprotective effect, at least in part, via direct chemical reaction with H_2_O_2_, albeit we did observe any visible reaction when these two chemicals were mixed at the concentrations used in current study. We speculate that one of the mechanisms underlying the protective effect of H_2_S on aberrant ENaC activity may be due to a direct chemical reaction of H_2_S with H_2_O_2_; and that this direct chemical reaction dramatically diminishes the ability of H_2_O_2_ to oxidize PTEN and subsequently enhances the apical distribution of PI(3,4,5)P_3_. Such direct chemical reaction has been reported by Geng *et al.*, where H_2_S directly scavenges superoxide anions and H_2_O_2_, and consequently eliminates ROS-induced malondialdehyde (MDA) generation [35].

Manna and Jain reported that H_2_S can increase cellular level of PI(3,4,5)P_3_ and can enhance glucose utilization in high concentration of glucose treated adipocytes by activating PI3K and inhibiting PTEN [14], [36]. These authors also demonstrated that there is a decreased cellular PI(3,4,5)P_3_ level and impaired glucose homeostasis in the liver of both type 1 and type 2 diabetic rats [36]. In contrast, our data suggest that NaHS did not affect total expression level of PTEN, but significantly prevented H_2_O_2_-induced inactivation of PTEN, thereby reversed H_2_O_2_-induced accumulation of PI(3,4,5)P_3_ near the apical compartment of A6 cells. The difference between the results obtained by Manna *et al.* and ours might be explained by cell model-dependence. Nevertheless, diabetes is associated with lower circulating level of H_2_S compared to normal population [30], [37], [38] and diabetes exerts a higher incidence of hypertension. Our results along with the findings by Manna and Jain suggest that H_2_S may be used for treatment of diabetes associated hypertension by affecting cellular level and distribution of PI(3,4,5)P_3_.

Oxidation of PTEN by ROS/H_2_O_2_ has been reported in HEK293 cells stimulated with insulin [9], HeLa cells stimulated with epidermal growth factor [9], and fibroblasts stimulated with platelet-derived growth factor [39]. It has been shown that PI(3,4,5)P_3_ can laterally diffuse in the inner leaflet of epithelial cell membranes from the basolateral membrane domain, where it is generated, to the apical membrane domain [40]. Theoretically, the apical PI(3,4,5)P_3_ would be rapidly degraded because the lipid phosphatase PTEN is mainly located in the apical membrane in epithelial cells [24]. H_2_O_2_ not only inactivates PTEN but also activates PI3K [6] which phosphorylates PI(4,5)P_2_ to produce PI(3,4,5)P_3_. NaHS prevents H_2_O_2_ induced inactivation of PTEN, but may not affect the PI3K activity. Therefore, it is not surprising that NaHS prevents accumulation of H_2_O_2_-induced PI(3,4,5)P_3_ specifically near the apical compartment but not in the lateral and basal membranes. Since only the levels of PI(3,4,5)P_3_ in the apical membrane are important to ENaC function, we have not quantified the fluorescent intensity of basal and lateral membranes. They may vary from cell to cell. Our data show that NaHS may not prevent accumulation of H_2_O_2_-induced PI(3,4,5)P_3_ in the basal and lateral membranes (Figure 3).

Leslie and co-workers demonstrated that oxidative inactivation of PTEN in the cells exposed to H_2_O_2_ is accompanied by an increase in cellular PI(3,4,5)P_3_ [41]. Furthermore, oxidation of PTEN with H_2_O_2_ leads to the formation of a disulfide bond between Cys124 residue and a nearby Cys71 residue in the active site, thus inactivating its phosphatase function of PTEN [10]. Oxidized PTEN has two less cysteine residues available for alkylation, which should lead to a lower molecular weight form of the protein. Consistent with this notion, we found that the abundance reduced (active) form of PTEN was dramatically reduced in A6 cells incubated with H_2_O_2_, whereas the magnitude of oxidized (inactivated) PTEN was significantly augmented in H_2_O_2_-treated A6 cells. However, there was no significant change in the abundance of total PTEN upon treatment A6 cells with H_2_O_2_. These results suggest that H_2_O_2_ inactivates PTEN by oxidizing its cysteine residues, which results in a defective hydrolysis of PI(3,4,5)P_3_. We suggest that the protective effect of H_2_S on aberrant ENaC activity caused by exogenous H_2_O_2_ is most likely due to the fact, that H_2_S can antagonize H_2_O_2_ mediated PTEN catalytic inactivation.

The importance of PTEN in regulating cellular distribution of PI(3,4,5)P_3_ is also supported by the results obtained by the pharmacological experiments, where application of BPV_(pic)_, a specific PTEN inhibitor, to the cells leads to accumulation of PI(3,4,5)P_3_ near the apical compartment of membrane and to an increase in ENaC *P_O_*. While the cells were incubated with both BPV_(pic)_ and H_2_O_2_, the abundance of PI(3,4,5)P_3_ near the apical compartment of the cell membrane and ENaC activity were increased. These synergistic effect of BPV_(pic)_ and H_2_O_2_ on ENaC and PI(3,4,5)P_3_ might be due to H_2_O_2_ -induced activation of PI3K.

Consistent with our previous findings [6], H_2_O_2_ not only increases ENaC *P_O_*, but also elevates ENaC single-channel amplitude. Interestingly, the effect of H_2_O_2_ on ENaC single-channel amplitude is also abolished by NaHS. The mechanism by which H_2_O_2_ elevates ENaC single-channel amplitude remains to be determined. We also noticed that treatment of the cell with H_2_S alone also slightly, but insignificantly decreased ENaC *P_O_*, this result suggests that slight oxidation stress may account, at least in part, for basal ENaC activity because A6 cells we used were cultured in the presence of aldosterone, which is known to elevate ROS in A6 cells [5]. However, H_2_S appears to be more important for strongly activated ENaC under oxidative stress. Since oxidative stress is common in salt-sensitive kidney [3], [4], further investigation of the effect of H_2_S on ENaC activity in animal models may benefit the clinical management of salt-sensitive hypertension.

## Supporting Information

Figure S1 **H_2_S does not affect cell viability.** Cell viability was estimated by MTT assays. NaHS at concentrations of 0.05, 0.1 and 0.3 mM had no effect on cell viability.
(TIF)

Figure S2 **Effect of 0.3 H_2_O_2_ on PI(3,4,5)P_3_ levels near the apical compartment of A6 cells.** Left images show confocal microscopy XY sections in the vicinity of the apical, lateral, and basal membranes of A6 cells as indicated. Right images show XZ sections of A6 cells. (A) Control; (B) A6 cells were treated with 0.3 mM for one hr. (D) Summarized mean fluorescent intensities measured in and in the vicinity of the apical membrane from three independent experiments, which represent the level of PI(3,4,5)P_3_ near the apical compartment of the cell membrane. *P<0.05 compared with control group.
(TIF)

Text S1 **MTT assay has been used to detect whether H_2_S affects viability of A6 cells.**
(DOC)
