# Correction: Hydrogen Sulfide Prevents Hydrogen Peroxide-Induced Activation of Epithelial Sodium Channel through a PTEN/PI(3,4,5)P_3_ Dependent Pathway

**DOI:** 10.1371/annotation/1cc3a25b-dc35-4d2a-8465-b11916f2e863

**Published:** 2013-10-17

**Authors:** Jianing Zhang, Shuo Chen, Huibin Liu, Bingkun Zhang, Ying Zhao, Ke Ma, Dan Zhao, Qiushi Wang, Heping Ma, Zhiren Zhang

The specification of recipients of funding were incorrectly omitted from the Funding Statement.

In addition, a funding organization and grant were incorrectly omitted from the Funding Statement.

The Funding Statement should read: "This study was supported by the Key Project of Chinese National Program for Fundamental Research and Development (973 Program, 2012CB517803), National Natural Science Foundation of China (81270340 and 81070217) to Z.R. Zhang, the Innovation Foundation of Harbin Medical University (YJSCX 2011-319HLJ) to J.N. Zhang, Postdoctoral Science Foundation of China (2012M520775) to H.B. Liu and Department of Health and Human Services (National Institute of Health Grant 5R01-DK067110) to H.P. Ma. The funders had no role in study design, data collection and analysis, decision to publish, or preparation of the manuscript." 

