# Supplementary figures and images for: Hydrogen Sulfide Prevents Hydrogen Peroxide-Induced Activation of Epithelial Sodium Channel through a PTEN/PI(3,4,5)P3 Dependent Pathway
Source: PLoS One. 2013 May 31;8(5):e64304. doi: 10.1371/journal.pone.0064304 (PMC3669336; doi:10.1371/journal.pone.0064304)

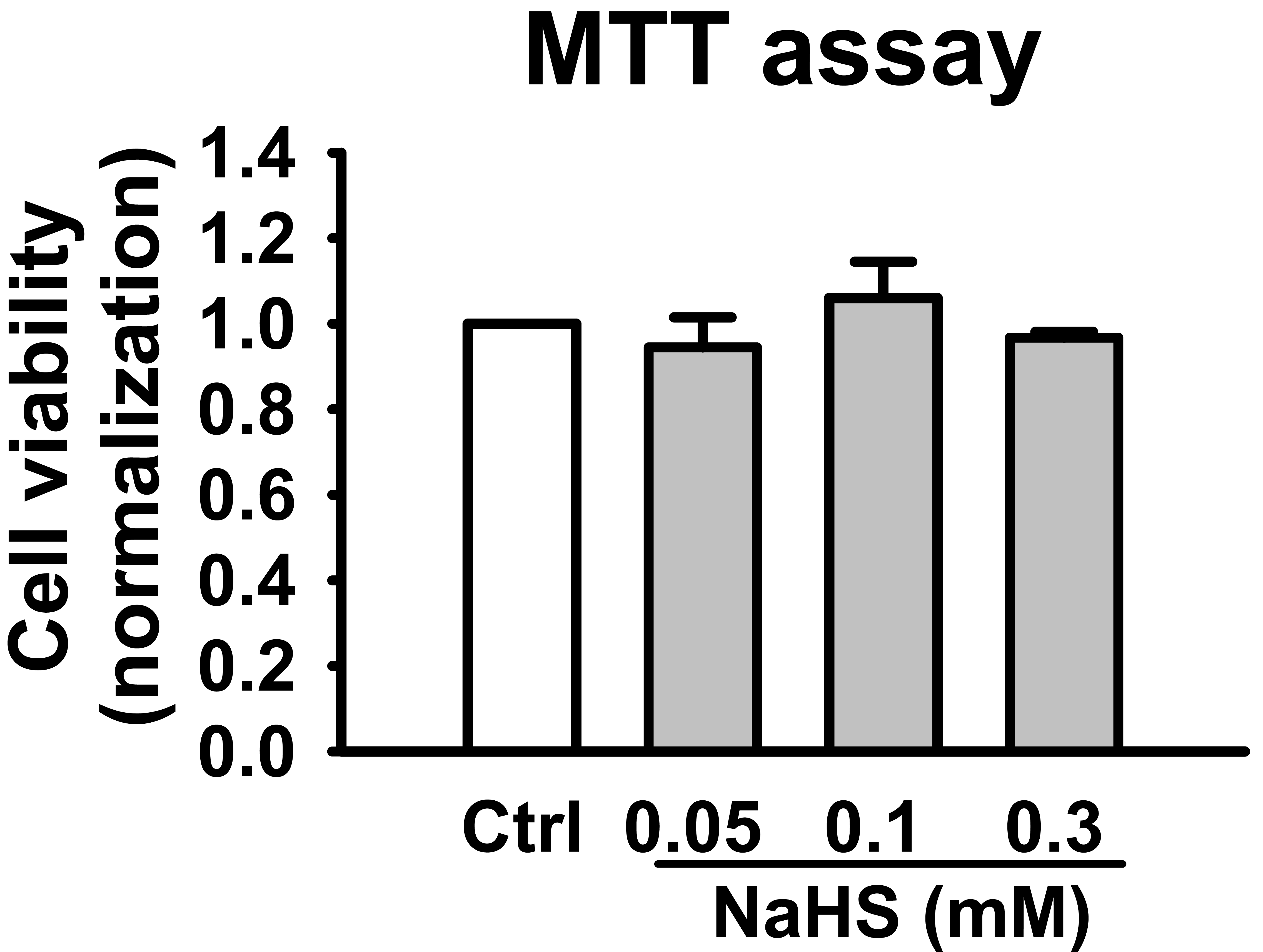

Supplement: Figure S1 — H2S does not affect cell viability. Cell viability was estimated by MTT assays. NaHS at concentrations of 0.05, 0.1 and 0.3 mM had no effect on cell viability. (TIF) [file pone.0064304.s001.tif]

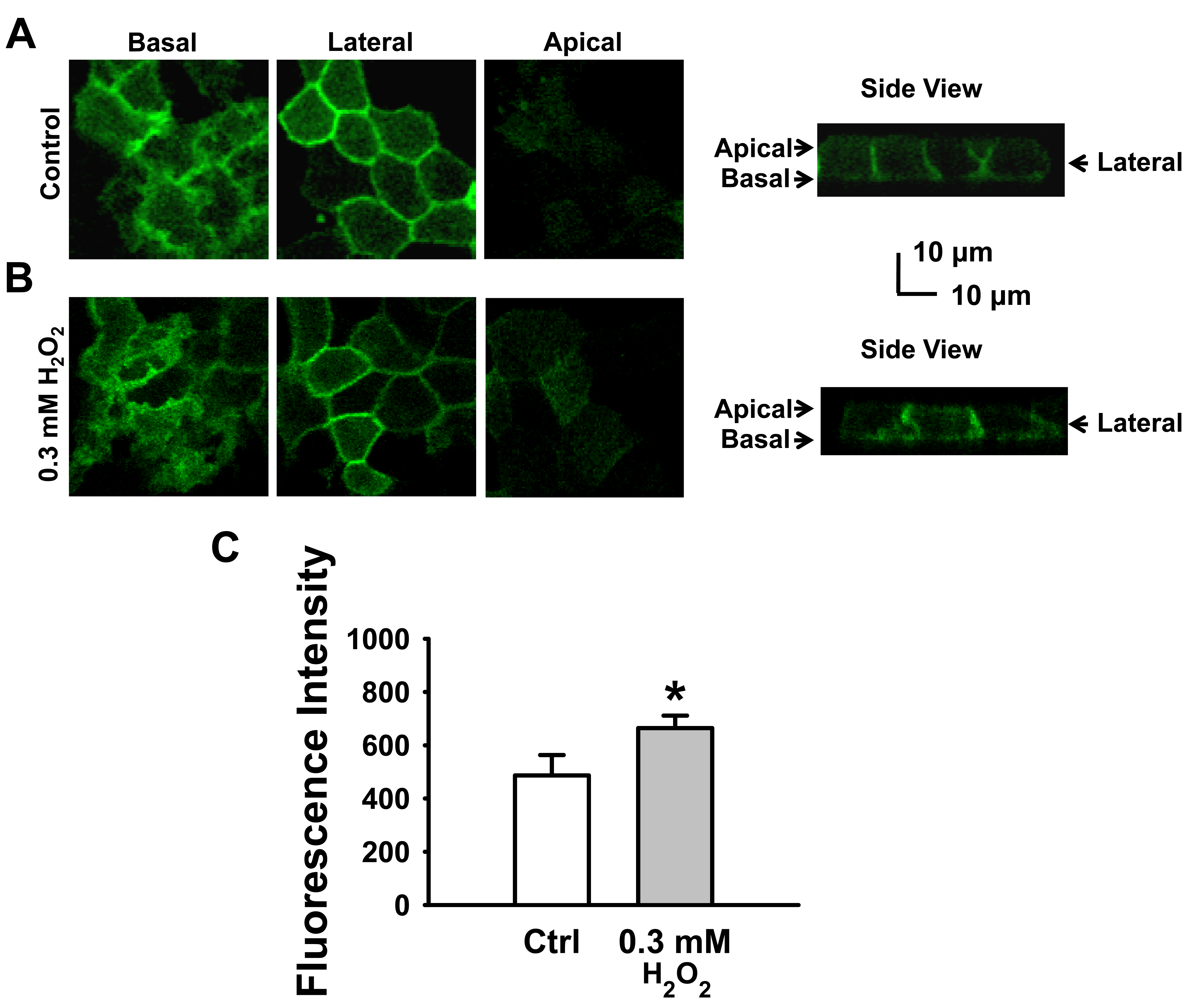

Supplement: Figure S2 — Effect of 0.3 H2O2 on PI(3,4,5)P3 levels near the apical compartment of A6 cells. Left images show confocal microscopy XY sections in the vicinity of the apical, lateral, and basal membranes of A6 cells as indicated. Right images show XZ sections of A6 cells. (A) Control; (B) A6 cells were treated with 0.3 mM for one hr. (D) Summarized mean fluorescent intensities measured in and in the vicinity of the apical membrane from three independent experiments, which represent the level of PI(3,4,5)P3 near the apical compartment of the cell membrane. *P<0.05 compared with control group. (TIF) [file pone.0064304.s002.tif]
