# Supplementary material for: Hydrogen Sulfide Prevents Hydrogen Peroxide-Induced Activation of Epithelial Sodium Channel through a PTEN/PI(3,4,5)P3 Dependent Pathway
Source: PLoS One. 2013 May 31;8(5):e64304. doi: 10.1371/journal.pone.0064304 (PMC3669336; doi:10.1371/journal.pone.0064304)
Supplement: Text S1 — MTT assay has been used to detect whether H2S affects viability of A6 cells. (DOC) [file pone.0064304.s003.doc]

**Cell viability assay**

Cell Viability was assessed by measuring mitochondrial dehydrogenase activity, using the colorimetric MTT assay, based on the fact that viable cells (but not dead cells) can reduce 3-(4,5-dimethylthiazol-2-yl)-2,5-diphenyl tetrazolium bromide (MTT)，as previously described in our previous works [1]. Cells were cultured in the 96-well culture plates and respectively treated with 0.05 μM, 0.1 μM and 0.3 μM NaHS for 30 min. Then, 100 μl MTT solution (0.05% MTT) was added to each well of the plat and the cells in the plat were incubated for four hr in the incubator. The absorbance at 450 nm was measured with a multifunctional ELIASA (Infinite M200, TECAN, Austria). Means of 4 wells optical density (OD) in the indicated groups were used to calculated specific ratio of cells viability according to calculated percentage of cells viability as follow：

Normalized cell viability = OD treatment group / OD control group.

**Reference**

1. Zhao D, Chu WF, Wu L, Li J, Liu QM, et al. (2010) PAF exerts a direct apoptotic effect on the rat H9c2 cardiomyocytes in Ca^2+^-dependent manner. Int J Cardiol 143: 86-93.
